# Low TIM3 expression indicates poor prognosis of metastatic prostate cancer and acts as an independent predictor of castration resistant status

**DOI:** 10.1038/s41598-017-09484-8

**Published:** 2017-08-21

**Authors:** Junlong Wu, Guowen Lin, Yao Zhu, Hailiang Zhang, Guohai Shi, Yijun Shen, Yiping Zhu, Bo Dai, Dingwei Ye

**Affiliations:** 1 Department of Urology, Fudan University Shanghai Cancer Center, Shanghai, 200032 China; 2 Department of Oncology, Shanghai Medical College, Fudan University, Shanghai, China

## Abstract

T cell immunoglobulin 3 (TIM3) is a cell surface star molecule expressed on T cells, and also marks dysfunctional CD8^+^ T cells in various kinds of cancers. However, there are few studies focusing on the expression of TIM3 in tumor cells. In our study, we recruited 139 patients with metastatic prostate cancer (mPCa) who received transurethral resection of the prostate (TURP) consecutively to examine whether TIM3 expression level is associated with overall survival (OS) in mPCa patients. Immunohistochemistry was performed to determine TIM3 expression in prostate cancer tissues and then patients were divided into two groups. In multivariate Cox analysis, we revealed that mPCa patients with negative TIM3 expression, younger age, no radiotherapy, higher Gleason score, higher cT stage and patients of mCRPC had a shorter OS. Therefore, a predictive nomogram was generated with identified independent prognostic factors to assess patients’ OS at 3 years. Multivariate logistic regression revealed that higher cT stage, higher Gleason score and low TIM3 expression were independent predictors of metastatic castration resistant prostate cancer (mCRPC). In conclusion, low expression level of TIM3 in prostate cancer tissues is an independent prognostic factor of poor prognosis for mPCa patients, and also an independent predictor of mCRPC.

## Introduction

Prostate cancer (PCa) is the most common non-skin cancer in men globally and the second leading cause of cancer-caused death in developed countries^1^. The incidence of PCa in China has increased rapidly over the past decade due to the increasing life expectancy, dietary changes and Westernized lifestyle^2, 3^. Although patients with localized PCa have a much favorable survival, metastatic prostate cancer (mPCa) remains a lethal disease^4^. Interestingly, a large proportion of newly diagnosed PCa patients already have metastatic disease because of a lack of prostate-specific antigen (PSA) screening and digital rectal examination in China^5^. To date, androgen-deprivation therapy (ADT) is the gold-standard therapy for advanced PCa patients^6^. Although response rates to ADT are almost 80%, most patients will eventually progress to castration-resistant status, which is accompanied by poor outcome and high lethality^7, 8^. Therefore, knowledge of novel biomarkers for predicting prognosis of mPCa is of great importance and interest.

T cell immunoglobulin-3 was thought to be a novel immune regulator. Initial examination revealed that TIM3 selectively expressed on the IFN-γ-producing CD4^+^ T helper 1 (Th1) and CD8^+^ T cytotoxic 1(Tc1) T cells and had negative regulative function of type 1 immunity^9^. In the field of anti-cancer research, co-blockade of the TIM3 and PD-1 pathways is superior to block PD-1 pathway alone at improving anti-tumor effect of the immune system, in both solid and hematologic cancer^10–12^. Maybe the function of TIM3 in tumor immunology is so highlighted that its effects in other fields are masked or ignored for a long time. Recently, prognostic values of TIM3 expression in tumor cells were investigated in colorectal cancer^13^, renal cell carcinoma^14^, gastric cancer^15^ and also localized prostate cancer^16^. However, the role of TIM3 expression in metastatic prostate cancer is still poorly understood.

In the present study, we want to find out whether TIM3 expression level is associated with the prognosis of mPCa patients and if it can serve as a biomarker to tell metastatic castration-resistant prostate cancer (mCRPC) from metastatic hormone-sensitive prostate cancer (mHSPC).

## Results

### Clinical characteristics of patients in our cohort

Of 139 patients in our cohort, the median age at TURP was 66 years old, ranging from 36 to 87 years old. Among all these patients, 45 persons had mCRPC while other 94 patients had mHSPC. In this database, median PSA at TURP was 71.08 ng/ml, ranging from 0.38 to 5000 ng/ml. Information of Gleason score, clinical T stage, N stage, androgen deprivation therapy, chemotherapy and radiotherapy was summarized in Table 1. The median follow-up time was 22.1 months among all these mPCa patients and 56 patients died during follow-up.

**Table 1:** Patient demographic and clinical characteristics.

Characteristics	Total cohort (N = 139) N (%)	TIM3 expression		P value
		Positive (N = 35)	Negative (N = 104)	
Age at TURP, Median (range)	66 (36–87)	64 (36–82)	68 (43–87)	0.270^a^
cT stage at TURP				0.056^b^
T2	59 (42.4)	15 (42.9)	44 (42.3)	
T3	24 (17.3)	8 (22.9)	16 (15.4)	
T4	24 (17.3)	9 (25.7)	15 (14.4)	
Tx	32 (23.0)	3 (8.6)	29 (27.9)	
cN stage at TURP				0.607^b^
N0	49 (35.3)	14 (40.0)	35 (33.7)	
N1	62 (44.6)	16 (45.7)	46 (44.2)	
Nx	28 (20.1)	5 (14.3)	23 (22.1)	
Gleason Score				0.392^b^
6	5 (3.6)	0 (0.0)	5 (4.8)	
7	18 (12.9)	6 (17.1)	12 (11.5)	
≥8	116 (83.5)	29 (82.9)	87 (83.7)	
PSA at TURP, Median (range)	71.80 (0.38–5000)	108 (3.39–1535)	62.06 (0.38–5000)	0.957^a^
mPCa subtype				**0.011** ^**b**^
mHSPC	94 (67.6)	30 (85.7)	64 (61.5)	
mCRPC	45 (32.4)	5 (14.3)	40 (38.5)	
ADT prior to TURP				0.497^b^
Yes	127 (91.4)	31 (88.6)	96 (92.3)	
No	12 (8.6)	4 (11.4)	8 (7.7)	
Chemotherapy				1.000^b^
Yes	44 (31.7)	11 (31.4)	33 (31.7)	
No	95 (68.3)	24 (68.6)	71 (68.3)	
Radiotherapy				1.000^b^
Yes	5 (3.6)	1 (2.9)	4 (3.8)	
No	134 (96.4)	34 (97.1)	100 (96.2)	

^a^Student’s t test. ^b^Fisher exact test (based on Chi-square). TURP: transurethral resection of the prostate. ADT: androgen-deprived therapy.

**Figure 1:**
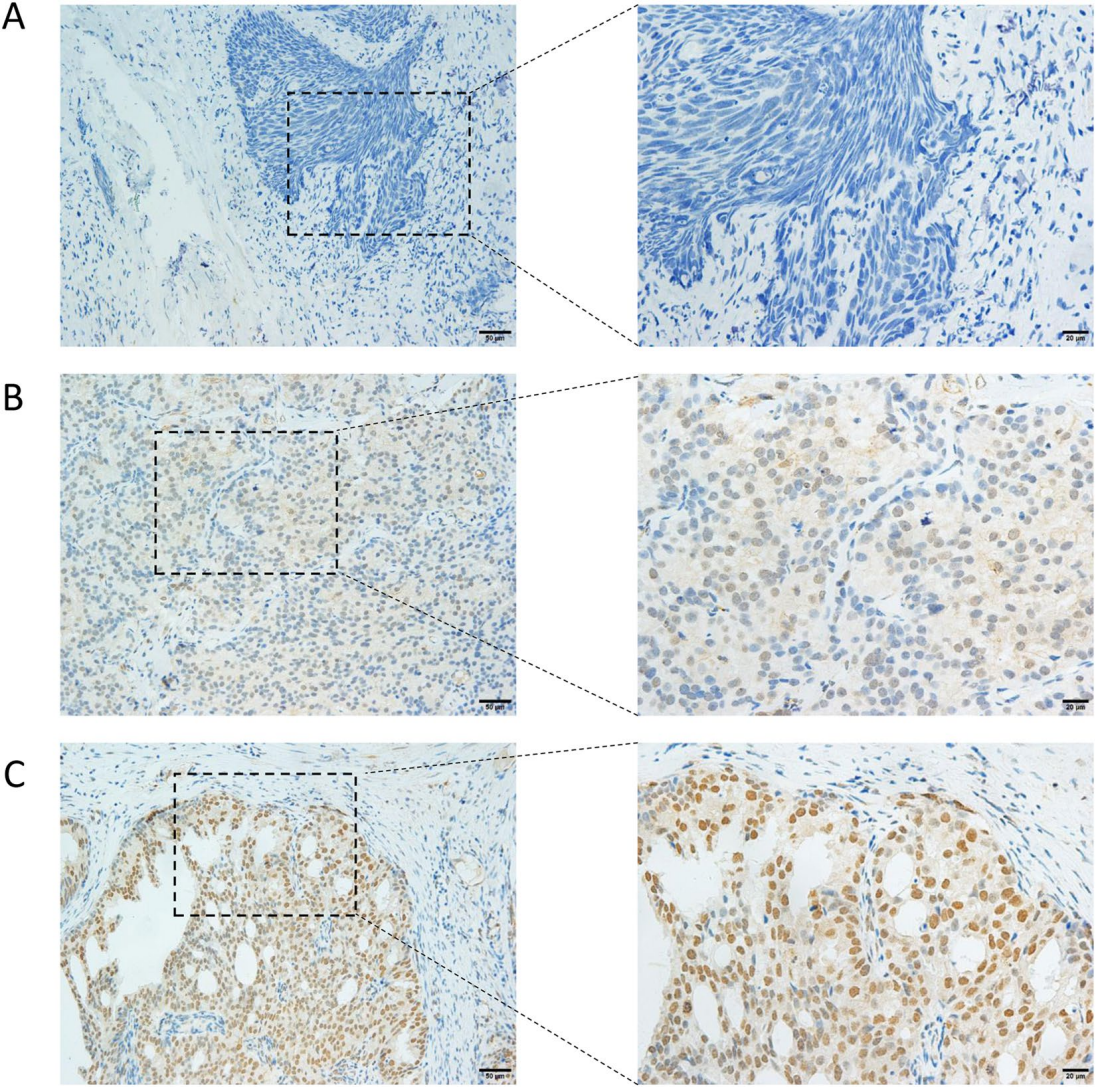
Representative images of immunohistochemical staining of TIM3 in prostate cancer cells. (**A**) No staining. (200X and 400X). (**B**) Weak staining. (200X and 400X). (**C**) Strong staining. (200X and 400X).

Representative images of immunohistochemical null, weak and strong staining of TIM3 in prostate cancer cells were shown in Fig. 1. The entire cohort was separated into positive TIM3 expression group (N = 35) and negative TIM3 expression group (N = 104) based on staining intensity and proportion as described. No significant difference in age, cT stage, cN stage, Gleason score, PSA, ADT status, radiotherapy and chemotherapy between these two subgroups defined by TIM3 expression was achieved. However, we noticed that in TIM3 negative group, the proportion of mCRPC patients was significantly higher than that in TIM3 positive group (Table 1). Quality of the antibody we used in this study was confirmed in bladder cancer, penile cancer, renal cell carcinoma and normal prostate acinar tissues (Supplementary Figure 1). In addition, TIM3 antibody from another commercial source (Cat. No. ab 185703, Abcam) was also used to verify the location of TIM3 in some metastatic prostate cancer tissues and normal prostate tissues (Supplementary Figures 2 and 3). These data supported our methodology and made our findings much more convincing.

### Negative TIM3 expression is an independent prognostic factor of poor prognosis in mPCa patients

**Figure 2:**
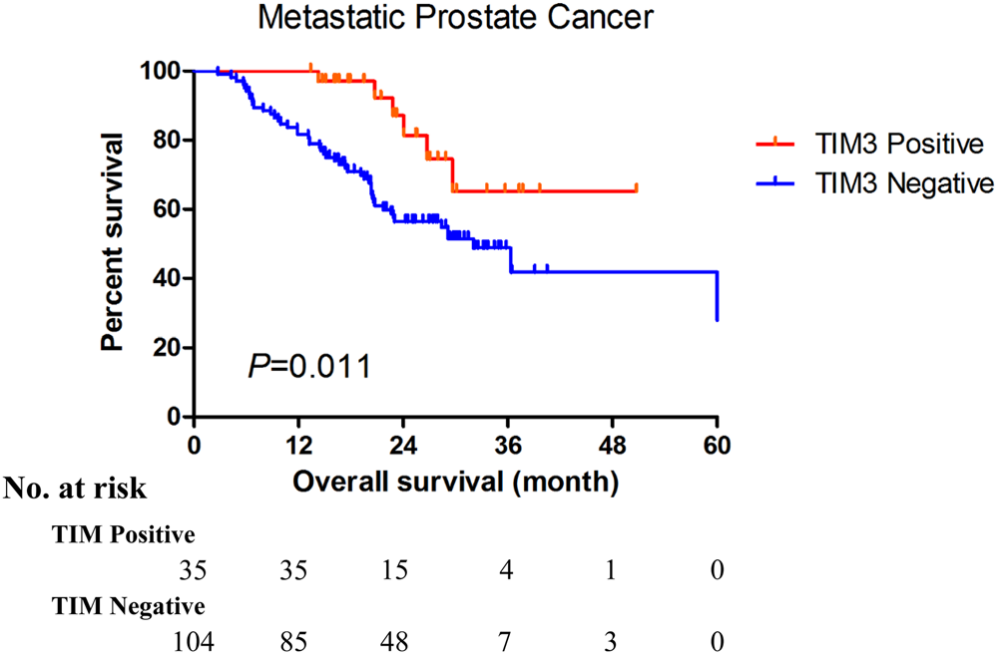
The Kaplan-Meier plot of overall survival in metastatic prostate cancer patients (according to TIM3 expression level).

To examine whether TIM3 expression level in prostate cancer cell has a prognostic value for overall survival in mPCa patients, a Kaplan-Meier curve of positive and negative TIM3 expression groups was depicted and presented in Fig. 2. In this plotting, patients with positive TIM3 expression had a significantly better prognosis (P = 0.011).

Furthermore, we generated univariate and multivariate Cox regression model in our database to investigate if TIM3 expression was an independent prognostic factor for predicting OS. In univariate Cox model, mCRPC patients (HR = 6.870, 95% CI: 3.872–12.190, P < 0.001), patients who received chemotherapy (HR = 2.506, 95% CI: 1.468–4.277, P = 0.001) and patients with negative TIM3 expression (HR = 2.865, 95% CI: 1.224–6.711, P = 0.015), higher cT stage (HR = 4.853, 95% CI: 2.365–9.960, P < 0.001), higher Gleason score (HR = 1.687, 95% CI: 1.240–2.295, P = 0.001) had significantly poorer prognosis (Table 2).

**Table 2:** Univariate and multivariate Cox regression analysis of overall survival in metastatic prostate cancer patients.

Characteristics	Univariate Cox analysis			Multivariate Cox analysis		
	HR	95% CI	P value	HR	95% CI	P value
Age at TURP	1.008	0.978–1.040	0.601	0.961	0.929–0.994	**0.021**
cT stage at TURP						
T2	Ref.			Ref.		
Otherwise	4.853	2.365–9.960	<**0.001**	2.981	1.381–6.435	**0.005**
cN stage at TURP						
N0	Ref.			Ref.		
Otherwise	2.326	1.199–4.512	**0.013**	1.215	0.538–2.747	0.639
Gleason Score	1.687	1.240–2.295	**0.001**	1.416	1.006–1.991	**0.046**
PSA at TURP	0.998	0.997–1.000	0.081	0.999	0.998–1.000	0.231
mPCa subtype						
mHSPC	Ref.			Ref.		
mCRPC	6.870	3.872–12.190	<**0.001**	5.222	2.711–10.058	<**0.001**
ADT prior to TURP				—	—	—
No	Ref.					
Yes	24.518	0.616–975.953	0.089			
Chemotherapy						
No	Ref.			Ref.		
Yes	2.506	1.468–4.277	**0.001**	1.688	0.933–3.055	0.084
Radiotherapy						
No	Ref.			Ref.		
Yes	0.763	0.181–3.218	0.712	0.157	0.030–0.831	**0.029**
TIM3 expression						
Positive	Ref.			Ref.		
Negative	2.865	1.224–6.711	**0.015**	2.976	1.182–7.519	**0.021**

Then multivariate Cox proportional hazards model was generated involving all the factors which were tested in univariate model, except ADT before TURP because it was highly associated with mPCa subtypes. With backward elimination, low TIM3 expression also remained statistical significance in predicting bad prognosis of mPCa patients (HR = 2.976, 95% CI: 1.182–7.519, P = 0.021). Besides, age at TURP, cT stage at TURP, Gleason score, mPCa subtype and radiotherapy were significantly associated with prognosis of mPCa patients in multivariate Cox analysis (Table 2).

### Extension of prognostic models with TIM3 expression for mPCa patients

To move forward a step, the prognostic power of TIM3 expression was assessed using C-index. As shown in Table 3, only including TIM3 expression in a prognostic model for OS, the C-index was 0.595 (95% CI: 0.552–0.637). Using other five independent factors which remained significant in multivariate Cox regression to generate a prognostic model, the C-index was 0.812 (95% CI: 0.758–0.867). Then, the C-index was improved from 0.812 to 0.827 (95% CI: 0.777–0.876) by adding TIM3 expression to build a new model, which showed a better predictive accuracy.

**Table 3:** Comparison of the accuracy of the prognostic models for overall survival.

Prognositic models for overall survival of mPCa patients	C-Index	95% CI
TIM3 Expression	0.595	0.552–0.637
mPCa subtype	0.730	0.688–0.772
Age	0.476	0.434–0.519
Gleason Score	0.612	0.570–0.655
Radiotherapy	0.502	0.459–0.544
cT stage	0.640	0.598–0.682
mPCa subtype + Age + Gleason Score + Radiotherapy + cT stage	0.812	0.758–0.867
TIM3 Expression + mPCa subtype + Age + Gleason Score + Radiotherapy + cT stage	0.827	0.777–0.876

**Figure 3:**
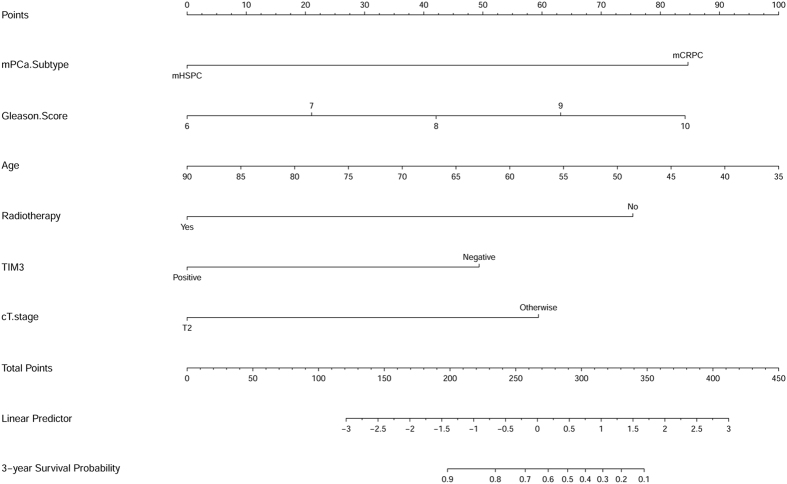
Nomogram for the prediction of 3-year overall survival after TURP in metastatic prostate cancer patients.

**Figure 4:**
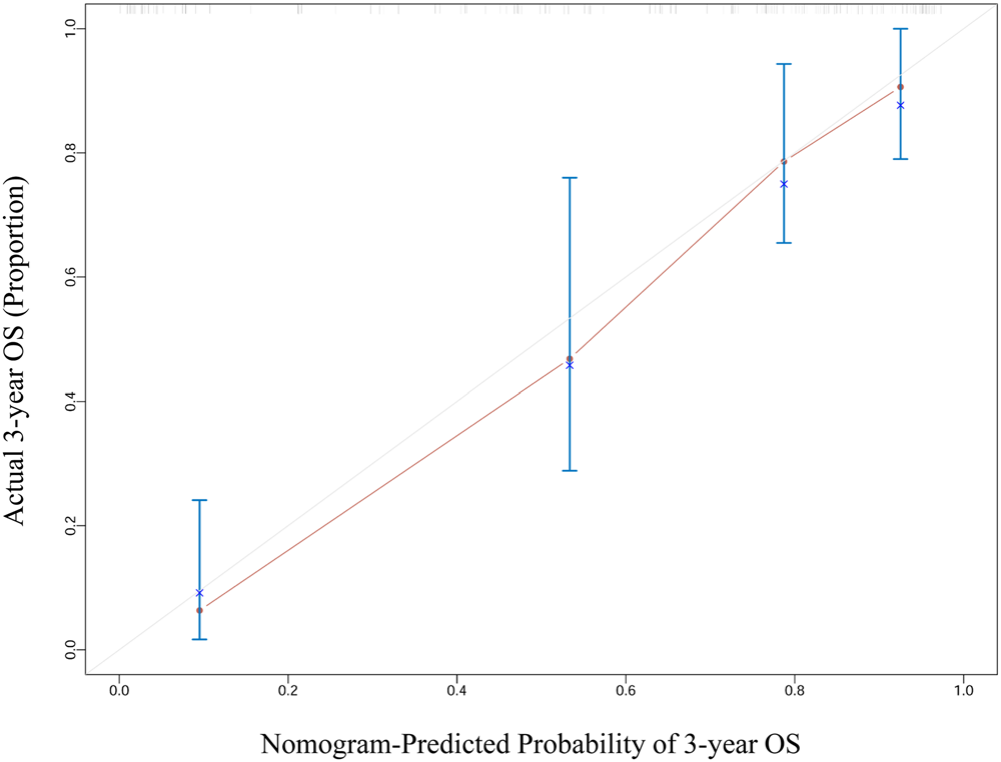
Calibration curve of the nomogram for the prediction of 3-year overall survival after TURP in metastatic prostate cancer patients.

Based on the results deriving from multivariate Cox regression of OS, along with the results of C-index analysis, we used data in our cohort to develop a nomogram to predict OS probability at 3 years after TURP (Fig. 3). In this plotting, we included TIM3 expression (positive vs negative), mPCa type (mCRPC vs mHSPC), age at TURP, cT stage at TURP, Gleason score and radiotherapy, which were all independent predictors for OS in multivariate Cox regression analysis. The calibration curve of nomogram was shown in Fig. 4.

### Negative TIM3 expression is an independent predictor of mCRPC

It really drew our interest that the proportion of mCRPC patients differed significantly between positive and negative expression groups. Univariate and multivariate logistic regression models were constructed to examine whether TIM3 expression had the ability to distinguish mPCa subtypes. In univariate logistic model, we found that cT stage over T2 at TURP (OR = 3.225, 95% CI: 1.462–7.114, P = 0.004) higher Gleason score (OR: 1.517 95% CI: 1.018–2.260, P = 0.041) and negative TIM3 expression (OR = 3.745, 95% CI: 1.344–10.417, P = 0.012) were considered to be a significant factor in predicting mCRPC (Table 4). Furthermore, in multivariate logistic model, negative TIM3 expression (OR = 3.448, 95% CI: 1.179–10.000, P = 0.024), higher Gleason score (OR = 1.557, 95% CI: 1.018–2.379, P = 0.041) and high cT stage (OR = 3.122, 95% CI: 1.362–7.155, P = 0.007) remained significant. So negative TIM3 expression may act as an independent predictor of mCRPC in metastatic prostate cancer patients.

**Table 4:** Univariate and multivariate Logistic regression analysis of predictors in predicting mCRPC.

Characteristics	Univariate Logistic analysis			Multivariate Logistic analysis		
	OR	95% CI	P value	OR	95% CI	P value
Age	1.032	0.988–1.079	0.155	1.023	0.974–1.073	0.362
cT stage at TURP						
T2	Ref.			Ref.		
Otherwise	3.225	1.462–7.114	**0.004**	3.122	1.362–7.155	**0.007**
cN stage at TURP						
N0	Ref.			Ref.		
Otherwise	2.097	0.947–4.644	0.068	1.319	0.485–3.588	0.588
Gleason Score	1.517	1.018–2.260	**0.041**	1.557	1.018–2.379	**0.041**
PSA at TURP	0.999	0.997–1.000	0.138	0.999	0.997–1.000	0.162
TIM3 expression						
Positive	Ref.			Ref.		
Negative	3.745	1.344–10.417	**0.012**	3.448	1.179–10.000	**0.024**

## Discussion

In our study, we were the first to demonstrate that TIM3 expression in local tumor cells was correlated with the prognosis of mPCa patients. In our cohort, we found that negative TIM3 expression was an independent prognostic factor of poor prognosis in mPCa patients. Nomogram to predict 3-year overall survival of mPCa patients was generated from this cohort, using all the independent prognostic factors including TIM3 expression. C-index and calibration curve of OS predictive model showed good concordance. In addition, we also found that negative TIM3 expression was an independent indicator of mCRPC.

Previously known, TIM3 was a protein mainly expressed on the cell surface, and its expression in T cells modulated immune response^17^. Increased TIM3 expression in monocytes/macrophages^18^, peripheral NK cells^19^, tumor infiltrating T cells^20, 21^ contributed to poor prognosis in various cancers. However, in our study, we found that low expression of TIM3 indicated a worse outcome. The possible mechanism explaining the discrepancy above might be that TIM3 expressed in tumor cells and tumor-associated immune cells had different tumorigenic patterns^22, 23^. Moreover, conflicting results concerning the association between TIM3 expression in tumor cells and survival in different cancers were generated. Positive expression of TIM3 in esophageal squamous cancer and bladder cancer indicated shorter survival^24, 25^, while elevated expression of TIM3 could be a protective factor in colon cancer cells^13^. Interestingly, TIM3 mainly expressed in the nucleus of colon cells, same as our detection in PCa cells. However, TIM3 expressed in the cell membrane and cytoplasm in bladder cancer cells and esophageal squamous cancer cells. This phenomenon may indicate that ectopic expression localization of TIM3 has totally different functions in tumor biology.

To find a prognostic factor for predicting survival in mPCa patients has become a hotspot for nearly a decade. Recent studies, while still somewhat limited, corroborated our results in some aspects. Rusthoven *et al*. found that lower GS was associated with improved overall survival, which was in accordance with our finding^26^. Clinical T stage and younger age were also considered to be independent prognostic factors of metastatic prostate cancer in several previous reports^27–29^. In the past 20 years, many studies convinced the finding and did further exploration, so as ours^30–32^, although there were also some conflicting results^26^. In this study, we found that low TIM3 expression in prostate cancer cells could be a predictor of poor prognosis in mPCa patients. despite the fact that the predictive strength of TIM3 was not that good like classic prognostic factors which were often used in clinical practice, such as mPCa subtype and cT stage, TIM3 as a biomarker, was significant and independent. This meant a lot because it might reveal a novel mechanism in mPCa progression, promote discovery of new therapy target and cast a new light into personalized management of mPCa patients.

Androgen deprivation therapy is the standard care for mPCa patients. However, nearly all mPCa patients will eventually progress to castration-resistant status. Although novel medicine like enzalutamide and systematic chemotherapy regimens had made great progress in treating mPCa, especially mCRPC patients, it was still far from ideal condition^33, 34^. Recently, some research groups focused on whether definitive treatment of the primary tumor can improve the prognosis of mPCa patients. Primary outcomes reported that radiotherapy to the primary tumor in addition to ADT could prolong survival of mPCa patients^35–38^. In our cohort, we only had five patients who received local radiotherapy. But the protective significance of radiotherapy still remained, which was an encouraging result. However, radiotherapy didn’t achieve statistical significance in univariate cox model. Relatively small sample size, selection bias and numerous confounders might explain this phenomenon. After adjustment, results from multivariate cox analysis should be more convincing.

The underlying mechanism for the transition from HSPC to CRPC has not been fully clarified. To date, studies in this field mostly focus on the AR-V7, which is deemed to be a novel marker and also plays an important role in AR signaling activation^39–41^. Potential mechanisms which were associated with androgen depletion-resistance included AR signaling activation^42, 43^, AR splicing variants^44, 45^ and PI3K/Akt/mTOR pathway activation^46^. In our cohort, we found that TIM3 expression in local tumor cells was not only an independent prognostic factor of mPCa, it could tell mHSPC from mCRPC as well. Concentrating more on whether nuclear expression of TIM3 in prostate cancer cells is linked with the pathways or molecules described above should be our next move.

Although the prognostic value and predictive ability of TIM3 expression in tumor cells of mPCa patients are revealed in our study, there are certainly some limitations. Firstly, this study has a retrospective nature and selected bias from a single cancer center. Secondly, although tissue cores from different areas were used to construct the TMA, the deviation of TMA analysis could not be avoided due to the heterogeneous nature of prostate cancer. Moreover, although the clinical significance of TIM3 expression was examined, the deep molecular role remained unknown. So in the future, we will expand our cohort to multicenter cohorts to further validate our primary outcome, and also do basic research to explore the deep mechanism behind the uplifting phenomenon we found.

In conclusion, negative TIM3 expression in local tumor cells is an independent prognostic factor of poor OS, and also an independent factor in predicting CRPC status in mPCa patients. Our finding updates the knowledge of predicting survival and hormone-sensitive to castration-resistant status transition in mPCa and also casts a new light in exploring new functions of TIM3.

## Methods

### Patients

We recruited 139 patients with metastatic prostate cancer who received transurethral resection of the prostate (TURP) in Fudan University Shanghai Cancer Center consecutively from 2008 to 2014. In these patietns, 45 of them were mCRPC patients while other 94 patients had mHSPC. CRPC was defined following EAU 2015 guideline: castrated serum testosterone level plus 3 consecutive rises in PSA 1 week apart resulting in two 50% increases over the nadir with PSA >2 ng/mL, or appearance of two or more new bone lesions on bone scan or enlargement of a soft tissue lesion. Prostate cancer tissues were obtained from TURP under an institutional review board-approved protocol. Each haematoxylin and eosin slide was reviewed by two pathologists independently to determine the presence of representative areas of original samples and to confirm tumor histology and Gleason score. Clinicopathological characteristics were obtained from our electronic records. Patients were followed up regularly by telephone or in the clinic once every three months.

Our presenting study was approved by the Institution Review Board of Fudan University Shanghai Cancer Center and met the ethical standards of the Helsinki Declaration II. Written informed consent was got from every patient enrolled.

### Tissue Microarray (TMA) and Immunohistochemistry (IHC)

We prepared TMA and performed IHC according to previously reported procedures^47^. Immunostaining of TIM3 was performed using a mouse monoclonal anti-TIM3 antibody (Cat. No. 60355-1-lg, Proteintech Group, Wuhan, China) and the Envision detection kit (Dako, Carpinteria, CA, USA). All IHC staining was independently assessed by two experienced pathologists. The staining intensity was graded from 0 to 2 (0, no staining; 1, weak staining; 2 strong staining) (Fig. 1). The staining extent was graded from 0 to 4 based on the percentage of immunoreactive tumor cells (0%, 1–5%, 6–25%, 26–75%, 76–100%). A score ranging from 0 to 8 was calculated by multiplying the staining extent score with the staining intensity score, resulting in a negative (0–4) staining or a positive (6–8) staining for each example. To confirm the quality of the TIM3 antibody we used in this article, we also used this antibody to detect TIM3 expression and location in other tissues which were previously reported with a specific sub-cellular location. In addition, we also used TIM3 antibody from another commercial source (Cat. No. ab 185703, Abcam, USA) to validate the location of TIM3 in some metastatic prostate cancer tissues and normal prostate acinar cells.

### Statistical analysis

Age at TURP and PSA level at TURP were considered as continuous variables and reported in the form of median (range). Clinical T stage, clinical N stage and Gleason score were counted as multiple categorical variables and shown as proportions. Metastatic prostate cancer subtype (mHSPC or mCRPC), receiving ADT before TURP or not, receiving chemotherapy or not, receiving radiotherapy or not and TIM3 expression were counted as binary variables and presented as proportions.

Overall survival (OS) was calculated from the date of TURP to the date of death or last follow-up. We used Kaplan-Meier method to construct survival curves and log-rank tests to assess differences between groups. Univariate and multivariate Cox analysis for mPCa patients were performed and adjusted hazard ratio (HR) was calculated based on Cox models. Nomogram for predicting 3-year OS was constructed with factors which maintained significance in multivariate Cox regression model included. Logistic regression models were developed to find independent factors which can tell mCRPC from mHSPC. Continuous data were compared with Student’s t test when the data matched the criteria of normal distribution. The Chi-square test was used to compare the distribution of categorical data between groups. All tests were two-tailed and P values less than 0.05 were considered to be statistically significant. SPSS software, version 22.0 (SPSS Inc., Chicago, IL, USA) and R software were used for data analysis and nomogram plotting.

## Electronic supplementary material

Supplementary Figure 1-3
