# Supplementary material for: Low TIM3 expression indicates poor prognosis of metastatic prostate cancer and acts as an independent predictor of castration resistant status
Source: Sci Rep. 2017 Aug 21;7:8869. doi: 10.1038/s41598-017-09484-8 (PMC5567055; doi:10.1038/s41598-017-09484-8)

# Title

# Low TIM3 expression indicates poor prognosis of metastatic prostate cancer and acts as an independent predictor of castration resistant status

Junlong Wu[1,2 #], Guowen Lin[1,2 #], Yao Zhu[1,2], Haliang Zhang[1,2], Guohai Shi[1,2], Yijun Shen[1,2], Yiping Zhu[1,2], Bo Dai[1,2,*], Dingwei Ye[1,2 *]

# These authors contributed equally to this work.

1. Department of Urology, Fudan University Shanghai Cancer Center, Shanghai, 200032, China
2. Department of Oncology, Shanghai Medical College, Fudan University, Shanghai, China

*Address for Correspondence: Prof. DingWei Ye, M.D. (dwyeli@163.com) and Dr. Bo Dai M.D. (bodai1978@126.com), Department of Urology, Fudan University Shanghai Cancer Center, No. 270 Dong'an Road, Shanghai 200032, People's Republic of China. Tel: 86-21-64175590-2807; Fax: 86-21-64434556;

# Supplementary File

## Supplementary figure legends:

**Supplementary Figure 1.** Representative images of TIM3 staining (60355-1-lg from Proteintech Group) in bladder cancer, clear cell renal cell carcinoma and penile cancer. TIM3 locates mainly in the membrane or cytoplasm in tumor cells of bladder cancer, clear-cell renal cell carcinoma and penile cancer, as previously reported. In addition, TIM3 locates on the membrane of normal prostate acinar cells (normal control). These data confirm the quality of the antibody we used in this study, and improve the reliability of our research.

**Supplementary Figure 2.** TIM3 antibody from another commercial source (Cat.No. ab 185703, Abcam, USA) was used as a control to further confirm the location of TIM3 in mPCa tissues. Positive staining of TIM3 locates in the nucleus (C) or both the cytoplasm and nucleus (B) using ab 185703 from Abcam. These results confirmed the location of TIM3 in tumor cells of mPCa patients.

**Supplementary Figure 3.** IHC staining was performed in normal prostate acinar tissues as a normal control with both antibody 60355-1-lg from Proteintech Group (A) and antibody ab 185703 (B) from Abcam. TIM3 locates in the membrane and cytoplasm in normal prostate acinar tissues.

**Supplementary Figure 1**

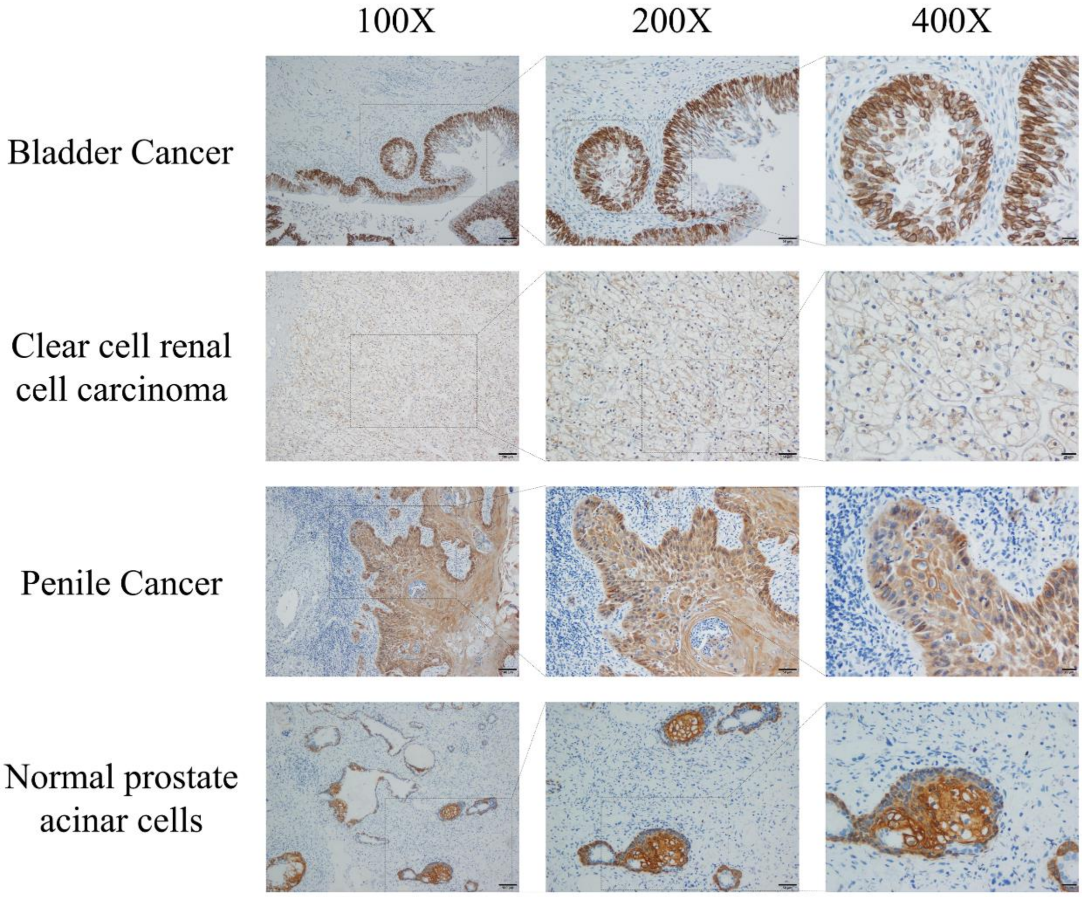

## Supplementary Figure 2

Cat. No. ab 185703 | 200X | 400X

A Negative Staining

B Positive Staining

C Positive Staining

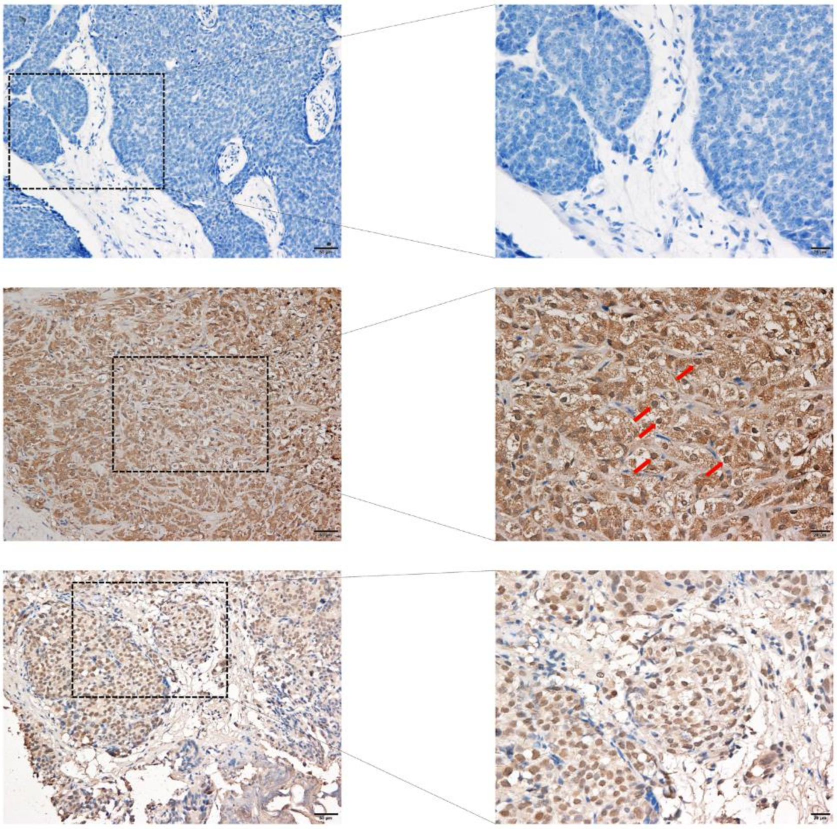

## Supplementary Figure 3

200X | 400X

A Normal prostate acinar cells

Cat. No. 60355-1-lg

B Normal prostate acinar cells

Cat. No. ab 185703

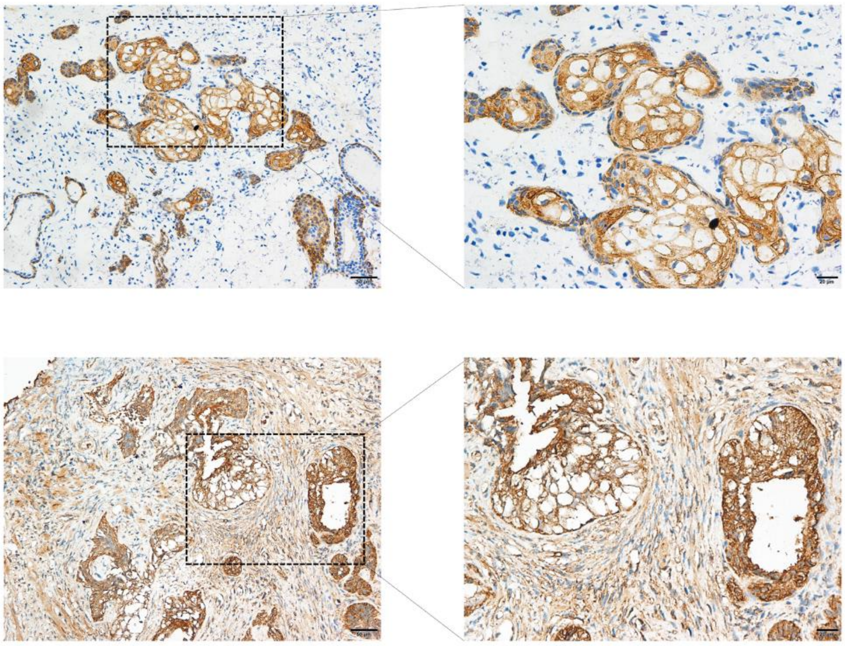

Supplement: Supplementary file 1 — Supplementary Figure 1-3 [file 41598_2017_9484_MOESM1_ESM.pdf]
